# Team-Based Professional Development Interventions in Higher Education: A Systematic Review

**DOI:** 10.3102/0034654317704306

**Published:** 2017-04-25

**Authors:** Inken Gast, Kim Schildkamp, Jan T. van der Veen

**Affiliations:** University of Twente

**Keywords:** professional development, higher education, team teaching, professional community

## Abstract

Most professional development activities focus on individual teachers, such as mentoring or the use of portfolios. However, new developments in higher education require teachers to work together in teams more often. Due to these changes, there is a growing need for professional development activities focusing on teams. Therefore, this review study was conducted to provide an overview of what is known about professional development in teams in the context of higher education. A total of 18 articles were reviewed that describe the effects of professional development in teams on teacher attitudes and teacher learning. Furthermore, several factors that can either hinder or support professional development in teams are identified at the individual teacher level, at the team level, and also at the organizational level.

Over the past 50 years, greater attention has been given to teacher professional development in higher education. Research on various types of professional development interventions has been published in this context, such as peer observations (e.g., Amrein-Beardsley & Osborn Popp, 2012; Chamberlain, D’Artrey, & Rowe, 2011), mentoring (e.g., Bryant-Shanklin & Brumage, 2011; Donnelly & McSweeney, 2011), courses or programs (e.g., Roberts & Weston, 2013; Skelton, 2013; Stenfors-Hayes, Weurlander, Dahlgren, & Hult, 2010), or the use of portfolios (e.g., Baume & Yorke, 2002; Fitzpatrick & Moore, 2013). Although recently more and more curriculum innovations in higher education involve teachers working together in teams, either to implement a curriculum innovation or to collectively improve their teaching knowledge and skills (e.g., Hoare et al., 2008; Lam & Tsui, 2014; Lefoe, Parrish, Keevers, Ryan, & McKenzie, 2013), professional development interventions are mostly individually focused. Therefore, there is a growing need for more research on professional development activities focusing on teams instead of only individual teachers.

In non–higher education contexts, much has been written about collaborating in teams or groups. There are various definitions of teams and groups, and in the literature, these terms are often used interchangeably. Our definition of a team is based on Katzenbach and Smith (1993, p. 112), who stated that “a team is a small number of people with complementary skills who are committed to a common purpose, performance goals, and approach for which they hold themselves mutually accountable.” In our article, the common purpose for a team is the design or implementation of a curriculum innovation in form of (re)design of a course or entire curriculum and/or the improvement of teaching. A pair of teachers working together and supporting each other is not considered a team for the purposes of this review. Work arrangements such as coteaching or mentoring relationships are therefore not included in this article.

Working in teams can be an effective method for professional development. West (1996) argued that team members bring different experiences to the table, which can be beneficial for the effectiveness of a team. West (1996) also stated that participating in a team creates commitment and reduced resistance to organizational change. According to Garet, Porter, Desimone, Birman, and Yoon (2001), professional development where teachers must collaborate with each other has several advantages. First, working together opens up opportunities to discuss problems, skills, and concepts. Second, teachers can share common materials. Third, teachers who share the same students can discuss student needs across classes or grade levels. Finally, working in groups creates a shared professional culture, and thereby helps sustain changes over time, for example, should teachers leave and new teachers join the faculty.

However, working in teams can also have some disadvantages, which need to be acknowledged. Research of Karau and Williams (1993) and Meyer, Schermuly, and Kauffeld (2016), for example, points to these disadvantages, such as social loafing (reduction in motivation and effort) when working in a team. Another problem of working in teams has been demonstrated by Kerr (1983), who researched the effect of free-riding on group efforts. Furthermore, LePine, Piccolo, Jackson, Mathieu, and Saul (2008) showed that there are several team processes that influence the effectiveness of a team, such as conflict management, monitoring the team’s progress, and specifying goals. Team members have to be cautious of these processes in order to perform well and reach their goals.

According to Guskey (2000), successful professional development activities must have an impact on teachers’ knowledge and skills, as well as teacher attitudes (see also Ajzen, 1991). Therefore, to ensure successful implementation of a team-based professional development intervention, a positive attitude toward the curriculum innovation or the teaching practice in question needs to be established, and teachers need to learn the knowledge and skills required to implement the curriculum innovation or the new practices. In non–higher education contexts, positive results for teacher professional development when working in teams have already been identified. Several studies have shown that teacher professional development in teams results in changes in teaching practice (e.g., Meirink, Imants, Meijer, & Verloop, 2010; Vescio, Ross, & Adams, 2008), new knowledge about teaching (e.g., Kafyulilo, Fisser, & Voogt, 2015), and changes in teachers’ attitudes (e.g., Meirink et al., 2010).

The goal of this study is to provide an overview of the effects of these team-based interventions on the professional development of teachers in the context of higher education. Furthermore, we also aim to identify various factors that might influence the successful implementation of these interventions.

There are various factors that can either hinder or support the professional development of teachers in teams (e.g., Binkhorst, Handelzalts, Poortman, & van Joolingen, 2015; Borg, 2012; Eameaim, Erawan, & Piromruen, 2009; Vangrieken, Dochy, Raes, & Kyndt, 2015). Studies have found several influential factors at the individual teacher level and the team level, as well as at the organizational level. Examples of factors at the teacher level include a positive attitude toward working in a team and knowledge of team processes (Vangrieken et al., 2015), as well as motivation to participate and the teachers’ experience (e.g., curriculum design experience; Binkhorst et al., 2015). Influential factors at the team level include trust between team members (Eameaim et al., 2009), team heterogeneity and team size (Vangrieken et al., 2015), and leadership (Borg, 2012). Finally, organizational factors that influence the success of teams include support from the school (Binkhorst et al., 2015; Vangrieken et al., 2015) and the school culture (Vangrieken et al., 2015). However, these factors were all identified in studies focusing on secondary education. The success-related factors for team-based professional development in higher education are still largely unknown. The time has come to review the evidence from higher education with regard to the effects of professional development in teams on teachers’ attitudes and learning. For this review, attitude is defined as “the degree to which a person has a favorable or unfavorable evaluation or appraisal of the behavior in question” (Ajzen, 1991, p. 188); in this study, the behavior in question is the curriculum innovation and/or new teaching practice. Following Guskey’s (2000, p. 121) description, we define teacher learning as “new knowledge and skills gained with regard to design and implementation of curriculum innovations and/or teaching practice.”

## Research Questions in the Higher Education Context

The context of higher education differs from other educational contexts. We should therefore not just accept the findings from research in primary and secondary education for the higher education context, but have to research whether these findings hold in higher education as well. One example for the differences in primary and secondary education and the context of higher education is that instructors at the university level are often not trained teachers but are scientists who are required to spend a part of their time on teaching. At research-oriented universities, in particular, staff evaluations are based on scientific output and not so much on their teaching performance (Graham, 2015).

Furthermore, working in teams is a fairly new concept for higher education teachers. The new educational trends in higher education, such as MOOCs (massive open online courses) or interdisciplinary courses, call for close collaboration between teachers in order to fulfill the organizational and teaching demands of these educational innovations. Percy and Beaumont (2008) argued that teaching teams in higher education are “the most logical and powerful site for addressing both the imperative for supporting the professional learning of . . . teaching staff and the concern for enhancing quality teaching and learning” (p. 152).

Although working in teams is becoming more and more important in the context of higher education, most studies published on teacher professional development in higher education have focused on individual professional development interventions (Stes, Min-Leliveld, Gijbels, & Van Petegem, 2010). Team-based professional development interventions have generally been neglected thus far. A systematic review was conducted to provide an overview of what is known about team-based professional development in higher education. The research questions guiding the search were the following: What are the benefits of team-based professional development in higher education in terms of (a) teacher attitudes and (b) teacher learning, and (c) under what conditions are these interventions most successful?

**Figure 1.**
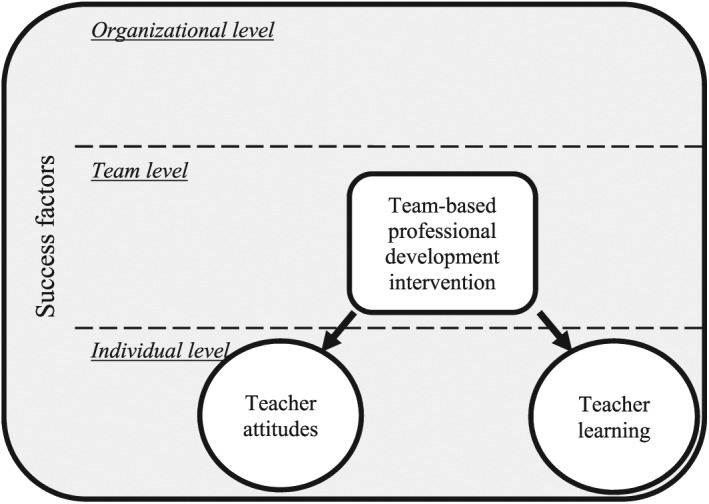
Conceptual framework for the review.

The conceptual framework guiding our review study is shown in Figure 1. This framework depicts teacher attitudes and teacher learning as dependent variables in team-based professional development interventions, which are influenced by a number of factors associated with the success of the intervention, at the individual, team, and organizational levels. Although, in the figure, teacher attitudes are depicted as an outcome of professional development interventions, they could also be a success-related factor at the individual teacher level. Furthermore, it is possible that success-related factors at all three levels might not only influence teacher attitudes and teacher learning but could also affect each other.

## Method

### Literature Search Procedure

The method used in this review is based on Petticrew and Roberts’s (2006) method for executing systematic reviews in the social sciences. This procedure involves several steps. First, research questions were formulated. Second, the search terms were defined and appropriate databases were selected. Third, inclusion and exclusion criteria were formulated, which further guided the literature search. Fourth, the scientific quality of the publications obtained was evaluated using predefined quality criteria. Only studies that met the quality requirements were included in this review. Finally, data answering the research questions were extracted.

### Databases and Literature Search Terms

A systematic review was conducted using four scientific databases: ERIC, PsycInfo, Scopus, and Web of Science. These databases were chosen due to their variety of journals involving educational research. Several combinations of search terms where used. We started with a combination of “professionalization,” “teacher,” and “higher education.” However, the term professionalization was not commonly used in the type of articles we were looking for in our review. We therefore removed that term and substituted the terms “professional development” or “professional learning community.” In the next step, we also used the combinations “professional development,” “team,” and “higher education.” After that, we used the term “team teaching” combined with “higher education.” Finally, we also used the combination of “community of practice,” “professional development,” and “higher education,” as this type of team is often referred to in literature and was not covered by the previous search queries. To gain a comprehensive overview of the articles on team-based professional development interventions in higher education, the search was not limited to a specific time span. However, only articles published in scientific peer-reviewed journals were included in this review; other scientific publications such as books or book chapters and conference papers were excluded. Furthermore, the articles had to be published in English and had to be available as a full-text version. Our search strategy resulted in a total of 914 publications.

### Selection Process

The abstracts of all publications obtained with the search terms described above were read and several inclusion criteria were applied:

- Only studies that were conducted in the context of higher education and had a team component were included in the review. This means that we included studies of various types of teams, such as teacher design teams, professional learning communities and the like, as well as articles that studied a formal professional development program that included a team component.
- The teams described in these articles had to have a common purpose of either collectively designing or implementing a curriculum innovation, or the goal of improving their own teaching.
- The articles had to provide information about teacher learning or teacher attitudes as a result of a professional development intervention and/or had to study conditions for successful team-based professional development interventions. We defined teachers as faculty members, who teach and/or design a course at a university. We perceived a professional development intervention to be successful when the participants had either gained new knowledge or skills or had improved their attitudes due to their participation.

After applying these inclusion criteria to all abstracts, 89 articles were selected for further analysis. The inclusion criteria were again applied to the full-text versions of the remaining articles, which resulted in a total of 28 articles that were selected for the final quality check.

### Initial Data Extraction and Quality Check

While reading the full-text versions of the articles, relevant data were extracted to evaluate the scientific quality of the studies described. The data extraction form that was used included the following sections:

1. *General information*: Study title, author, year of publication, country, research context, and journal
2. *Topic*: Teaching domain of teachers involved and purpose of the intervention described
3. *Research design*: Research question or research objective, description of the study, research design, research method, length of the intervention, and data analysis method
4. *Research population*: Number of respondents, gender, teaching experience, and sampling method
5. *Overall results*: Findings related to any one of the three parts of the research question

All 28 articles that were not excluded from the review due to the inclusion criteria had to pass a quality check. The quality of the articles was checked using 11 quality criteria drawn from Petticrew and Roberts (2006; see Table 1).

**Table 1:** Quality criteria

Category	Quality criteria
General	1. Is the research objective clear?
	2. Is the research done using the chosen method capable of finding a clear answer to the research question?
Selection sample	3. Were enough data gathered to assure the validity of the conclusions?
	4. Is the context of the research clear (country, participants)?
Method	5. Do the researchers state the research methods used?
	6. Do the authors give an argument for the methods chosen?
	7. Do the researchers take into account other variables that might be of influence?
Data analysis	8. Are the data analyzed in an adequate and precise way?
	9. Are the results clearly presented?
	10. Do the researchers report on reliability and validity of the research?
Conclusion	11. Is the research question answered using empirical evidence from the research that was done?

Each criterion was evaluated on a 3-point scale: 0, 0.5, or 1 point. To be included in the review, articles had to have a combined score of at least 5.5 for the 11 criteria, at least half of the maximum amount of points possible. To ensure that only articles with acceptable quality were selected and that other potentially suitable articles were not lost due to rater bias, a second rater did an independent scoring of 15% of the quality checks. Interrater reliability was evaluated by comparing both raters’ ratings on all criteria; an intraclass correlation of .78 was found. After this quality check, 18 articles remained that were eligible for inclusion in our review; 10 articles did not meet the quality criteria.

### Team-Based Professional Development Interventions: A Young Field of Study

**Figure 2:**
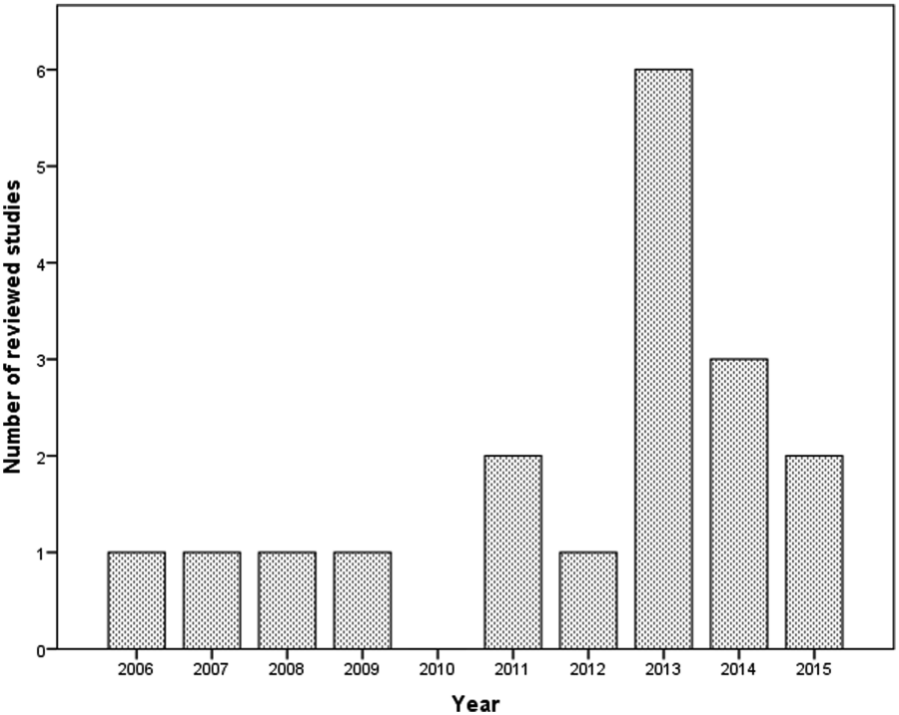
Frequency distribution for year of publication for reviewed articles.

When looking at the publication dates of the 18 articles reviewed in this study (see Figure 2), it becomes obvious that all articles were published during the past decade, with most of them published after 2010. This finding supports the contention that research on team-based professional development interventions in higher education is a fairly new field of study. In Table 2, an overview is given of the articles included in this review. Although only 18 eligible studies were found, it can be seen that studies on professional development in teams in higher education have been conducted all over the world. Furthermore, most of the studies reviewed made use of qualitative methods such as interviews, observations, and document analyses. There was also great variety in the types of teams studied, including, for example, communities of practice, teacher design teams, and teacher inquiry communities. Although some teams had a clear design focus and other teams focused more on teacher learning, they are all relevant for our review. Finally, only one of the articles included in this review specifically mentioned the term “attitudes” as a result of a team-based professional development intervention. Therefore, there was not enough evidence to draw conclusions about teacher attitude changes as an outcome variable of team-based professional development interventions. Thus, attitudes as an outcome of a team-based professional development intervention was dropped from further analysis.

**Table 2:** Overview of articles on team-based professional development interventions in higher education included in this review

No.	Article	Country	Intervention type^a^	Team purpose	Intervention length	Participants (*N*)	Research design	Research method
1	Bakah, Voogt, and Pieters (2012)	Ghana	Teacher design teams	Curriculum innovation	N/A	*N* = 63, plus leaders (29 teachers from design teams, 34 teachers not part of teams)	Mixed	I, FG, S
						Engineering faculty		
2	Blanton and Stylianou (2009)	USA	Community of practice	Teacher learning	2 Years	*N* = 11 (Year 1)	Qual	I, RE
						*N* = 12 (Year 2)		
						Experienced teachers		
						Mathematics department		
3	Bryant, Niewolny, Clark, and Watson (2014)	USA	Collaborative and interdisciplinary teaching team	Curriculum innovation	N/A	*N* = 11	Qual	FG
						Various levels of experience		
						Various disciplines		
4	Deni and Malakolunthu (2013)	Malaysia	Inquiry community	Teacher learning	1 Year	*N* = 8–10All femaleLanguage-based subjects	Qual	O, RE, QS, FG/I
						Various levels of experience		
5	Dickerson, Jarvis, and Levy (2014)	UK (England)	Project team blended learning	Curriculum innovation	10 Months	*N* = 8 (7 staff, 1 student)	Qual	I/S, D
6	W. Green, Hibbins, Houghton, and Ruutz (2013)	Australia	Community of practice	Teacher learning	≈6 Years (start in 2007)	*N* = 15	Qual	I
						Multidisciplinary		
						Various levels of experience		
7	Harwood and Clarke (2006)	UK	Teaching team	Curriculum innovation, teacher learning	3 Years	*N* = 43 (Survey 1)*N* = 38 (Survey 2)Marketing departmentVarious levels of experience	Mixed	S/QS, OD, D, R
	
	
	
8	Houghton, Ruutz, Green, and Hibbins (2015)	Australia	Community of practice	Teacher learning	≈8 Years (start in 2007)	*N* = 15	Qual	I
						Interdisciplinary business faculty		
						Experienced teachers		
9	Keevers et al. (2014)	International team: Australia and Malaysia	Transnational teaching team	Curriculum innovation	N/A	*N* = 60 (survey)	Mixed	I, S, O, RE
						*N* = 25 (interviews)		
						*N* =39 (practice development program)		
						Various levels of experience		
10	Kosnik et al. (2015)	Canada, USA, England, and Australia	Various	Teacher learning	N/A	*N* = 28	Qual	I
						Various levels of experience		
						Literacy/English teacher educators		
11	Margalef García (2011)	Spain	Teacher learning community	Teacher learning	N/A	*N* = 6 teams (at least 3 teachers per team); 2 health science teams, 1 social science team, 1 engineering team, 2 interdisciplinary teams	Qual	O, I, FG, L, FS
12	Margalef García and Roblin (2008)	Spain	Project team	Curriculum innovation, teacher learning	N/A	*N* = 6 (5 teachers, 1 PhD student as critical friend)	Qual	I, O
						Psychology/pedagogy faculty		
13	Norton, Russell, Wisner, and Uriarte (2011)	USA	Participatory action research team	Teacher learning	½ Year	*N* = 4	Qual	D, N
						Age: 30–50 years		
						Social work faculty		
						Various levels of experience		
14	Poyas and Smith (2007)	Israel	Community of practice	Curriculum innovation, teacher learning	2 Years	*N* = 30 (survey)	Mixed	I, S, R
						*N* = 6 (interviews and reflections)		
						Various levels of experience		
						Various domains		
15	Rienties, Brouwer, Bohle Carbonell, et al. (2013)	Netherlands	Online training	Teacher learning	8–12 Weeks	*N* = 65 (pretest)	Quan	S
						*N* = 36 (posttest)		
						Average age: 41.04 years; 58% male		
						Various levels of experience		
						Wide range of disciplines		
16	Rienties, Brouwer, and Lygo-Baker (2013)	Netherlands	Online training	Teacher learning	8–12 Weeks	*N* = 33	Quan	S
						Average age 41.90 years; 55% male		
						Mostly Dutch		
						Various levels of experience		
						Wide range of disciplines		
17	Roblin and Margalef (2013)	Spain	Inquiry community	Teacher learning	1 Year	*N* = 6 (5 teachers, 1 PhD student as critical friend)	Qual	I, O, N, RE, D
						Various levels of experience		
						Educational psychology faculty		
18	Schuck, Aubusson, Kearney, and Burden (2013)	Australia	Professional learning community	Teacher learning	1½ Years	*N* = 9, and 1 critical friendVarious levels of experienceTeacher education	Qual	D, OD, R

*Note*. N/A = not available.

a. Types of interventions as identified in the articles: Mixed = mixed methods; Qual = qualitative methods; Quan = quantitative methods; D = documents; FG = focus groups; FS = facilitator seminar; I = interviews; L = literature; N = narratives; O = observations; OD = online documents; QS = qualitative survey; R = reflections; RE = recordings; S = survey.

### Data Analysis

After having extracted the overall results from the articles, the results and discussion sections of all articles that met our quality criteria were analyzed in more detail to ensure a comprehensive portrayal of the results. Analysis of the results of these articles involved carrying out a thematic synthesis (see Thomas, Harden, & Newman, 2012). The results and discussion sections of all articles that met our quality criteria were coded line by line using ATLAS.ti. Inductive coding was used to identify various themes that were used to compare the articles according to their different results regarding teacher learning, and success-related factors for team-based professional development interventions. By comparing these articles, categories involving results related to several different aspects of teacher learning, as well as a number of success-related factors for team-based professional development interventions.

## Results

In this section, our findings in relation to the research questions are presented. We have subdivided the results into several themes. An overview of these themes and the supporting studies can be found in Table 3.

**Table 3:** Overview supporting studies per theme

Theme	Supporting studies
Learning	
Collegiality	5, 9, 10, 13, 14, 16
Critical reflection	2, 6, 11, 12, 14, 17, 18
Teaching approach	4, 5, 6, 7, 13, 14, 15, 16, 18
Pedagogical knowledge	7, 13, 14, 15, 16
Teacher identity	4, 6
Influential factors	
Individual level	
Attitudes	1, 11
Motivation	1, 2, 3
Commitment	3, 7, 12
Self-efficacy	11, 12, 18
Professional identity	2, 12
Availability	1, 5
Team level	
Team interaction	3, 5, 7, 8, 9, 11, 13, 18
Goals and objectives	11, 17, 18
Team composition	2, 4, 5, 6, 9, 10, 11, 18
Team leadership	5, 7, 11
Small group work	5, 14
* *Organizational level	
Organizational support	1, 4, 5, 7, 8, 9, 10, 11, 12, 14, 16, 18
Rewards	1, 2, 3, 5, 8, 9
Research focus	3, 16
Finances and resources	3, 10

*Note*. The numbers mentioned here refer to the identifying numbers in Table 2.

### Effects of Professional Development Interventions on Teacher Learning

The following section presents an overview of the effects on teacher learning due to teachers’ participation in team-based professional development interventions, from the studies reviewed. Only learning results that were at least described in two articles were included in this review. The articles included in this review reported on various kinds of learning results such as a change in pedagogical knowledge, teaching approach, and teacher identity.

#### Collegiality

Six articles described how participating in a team-based professional development intervention resulted in colleagues learning more about each other (Dickerson et al., 2014; Keevers et al., 2014; Kosnik et al., 2015; Norton et al., 2011; Poyas & Smith, 2007; Schuck et al., 2013). A participant in the qualitative study by Keevers et al. (2014) described how it is useful to get to know people you are working with, to empathize with the challenges they are going through. Using a mixed-methods approach, Poyas and Smith (2007) were able to show that the participants became more aware of similarities and common interests in their own work and the work of their colleagues due to their participation. In the study by Norton et al. (2011), teachers even became role models for each other.

#### Critical Reflection

Through participating in a team-based professional development intervention, teachers in higher education can learn to reflect critically on their own teaching practices. Seven articles reported about this critical reflection (Blanton & Stylianou, 2009; W. Green et al., 2013; Margalef García, 2011; Margalef García & Roblin, 2008; Poyas & Smith, 2007; Roblin & Margalef, 2013; Schuck et al., 2013). For example, Roblin and Margalef (2013) stated that the inquiry community they studied “enabled critical reflection that challenged teachers to make their educational beliefs explicit and to critically analyze the outcome of diverse activities” (p. 28). Acknowledging interpersonal and intrapersonal dilemmas experienced in inquiry communities and taking a critical perspective on own practices and beliefs was said to enable this critical reflection. However, critical reflection cannot always be achieved. Margalef García (2011) described that true reflection is often not undertaken, as old practices are replaced by different practices without changing one’s underlying conceptions.

#### Teaching Approach

Another learning outcome of participating in a team-based professional development intervention was a change in a participant’s teaching approach (Deni & Malakolunthu, 2013; Dickerson et al., 2014; W. Green et al., 2013; Norton et al., 2011; Rienties, Brouwer, & Lygo-Baker, 2013). For example, Deni and Malakolunthu (2013) concluded that teachers were readjusting their teaching style to more s*tudent-centered teaching or a democratic teaching style* with explicit empathy for students, as a result of participating in a teacher inquiry community. Deni and Malakolunthu used data triangulation involving interviews, observations, audio recordings, and a qualitative questionnaire to validate their findings. However, in the quantitative study by Rienties, Brouwer, and Lygo-Baker (2013), teachers did not develop a more student-centered teaching approach as a result of a team-based professional development intervention, which could be due to the difference in focus (use of a specific type of technology) and approach (online professional development) of this study. However, Rienties, Brouwer, and Lygo-Baker (2013) did find that teachers showed a decreased belief in simple knowledge transmission and wanted to move away from teacher-centered learning.

Teachers also gained hands-on examples of how to implement *new teaching strategies and teaching methods* in the classroom. Some articles generally stated that teachers had learned effective practices without going into detail about it (Harwood & Clarke, 2006; Poyas & Smith, 2007); other articles provided concrete examples of these effective practices (Deni & Malakolunthu, 2013; Dickerson et al., 2014; Norton et al., 2011; Rienties, Brouwer, Bohle Carbonell, et al., 2013; Rienties, Brouwer, & Lygo-Baker, 2013; Schuck et al., 2013). For example, teachers in the qualitative study by Norton et al. (2011) involving document analysis and teacher narratives, learned to improve their *teacher–student relationships* by conducting one-on-one meetings during the semester. However, new practices could not always be transferred from one classroom to another (Deni & Malakolunthu, 2013).

Other learning outcomes involved *a better understanding of students* and how to support their learning (Deni & Malakolunthu, 2013; Dickerson et al., 2014; W. Green et al., 2013; Harwood & Clarke, 2006; Norton et al., 2011). Deni and Malakolunthu (2013) stated that teachers gained better understanding of how to handle difficult students, and address learning difficulties, and gained better understanding of how to support students’ educational aims. Finally, several articles described teachers’ experimentation with new ideas gained through participation in team-based interventions. Deni and Malakolunthu (2013) and W. Green et al. (2013) stated that participating in a teacher inquiry community resulted in experimenting with new ideas, and that participating in a community of practice resulted in *experimentation with innovative practices*.

#### Pedagogical Knowledge

Five articles included in this review stated that the participants in team-based professional development interventions gained new pedagogical knowledge (Harwood & Clarke, 2006; Norton et al., 2011; Poyas & Smith, 2007; Rienties, Brouwer, Bohle Carbonell, et al., 2013; Rienties, Brouwer, & Lygo-Baker, 2013). In the study by Poyas and Smith (2007), participants responded in a questionnaire that they had learned some new concepts, improved their understanding of already known concepts, and revisited concepts discussed during meetings. In the mixed-methods study by Harwood and Clarke (2006), the team approach used resulted in greater clarity of teaching and of learning goals, due to open communication within the team. Furthermore, Rienties, Brouwer, Bohle Carbonell, et al. (2013) and Rienties, Brouwer, and Lygo-Baker (2013) found in their survey studies that teachers participating in online training with a team component reported that they were significantly more confident about their overall technological pedagogical content knowledge and their use of technology-enhanced learning in the classroom after completing the training.

#### Teacher Identity

Participating in a team-based professional development intervention affected how team members perceived themselves and their role as a teacher. In the mixed methods study by Deni and Malakolunthu (2013), teachers reported that they gained better understanding of themselves as a teacher and their role in the classroom when they viewed their professional commitments from others’ point of view. Participants in the study by W. Green et al. (2013) stated during interviews that their understanding of what it means to be a university teacher had changed. Being part of a community of practice affected not only what the teachers did but also what kind of teacher they were. Teachers became more aware of the role they played in their students’ development and how to influence this development. They gained greater confidence and became more innovative in this regard. One teacher, for example, described that she or he had greater confidence because she or he felt that she or he had done something, which was perceived by others as worthwhile.

### Conditions for Successful Professional Development in Teams

The literature about teams in higher education identified several conditions for successful professional development in teams. In the following section, these conditions are organized in three groups: conditions at the individual teacher level, conditions at the team level, and conditions at the organizational level. Only conditions that were mentioned at least in two articles were included in this review.

#### Conditions at the Individual Teacher Level

Six conditions were found at the individual level: attitudes, motivation, commitment, self-efficacy, professional identity, and availability.

##### Attitudes

Although changing teacher attitudes can be an outcome of professional development interventions, teacher attitudes prior to the start of a professional development intervention can also be a success-related factor for these interventions. According to Bakah et al. (2012), teachers’ positive attitudes regarding design teams were an important condition for the sustainability of these teams. Furthermore, Margalef García (2011) stated, based on her qualitative study, that prior participation in formative professional learning activities was positive for the progress of the teacher team. She argued that formal trainings or seminars “made it possible [for teachers] to develop positive attitudes towards reflective practice, a greater willingness to continue learning and enquiring into the teaching practice and a certain sensitivity required in order to accept constructive criticism” (Margalef García, 2011, p. 146).

##### Motivation

The motivation experienced by participants with regard to the implementation of an innovation was also found to be influential for the success of a team-based intervention (Bakah et al., 2012; Blanton & Stylianou, 2009; Bryant et al., 2014). In the literature, supporting intrinsic motivation was favored above enhancing external motivation. According to Bakah et al. (2012), teachers participating in design teams as well as teachers who did not participate in design teams agreed in a survey that teachers in a design team should be motivated. However, most teachers in design teams stated that they should not be rewarded financially (external motivation) to increase motivation, because this could lead, among other things, to people participating in design teams only for the monetary gains. Furthermore, participants should not be discouraged from participating in teams by jeopardizing their tenure (Bryant et al., 2014). According to Bakah et al. (2012), giving participants the freedom to design and implement their own professional development programs within the design teams would motivate the participants as well as support the development of needed innovations. Motivating participants in this way hints at the support of intrinsic motivation.

##### Commitment

Along with being motivated to participate in a team-based professional development intervention, teachers also need to be committed to their team (Bryant et al., 2014; Harwood & Clarke, 2006; Margalef García & Roblin, 2008). However, Bryant et al. (2014) noted based on their focus group interviews that the level of commitment needed from team members depends on the level of collaboration within a team. They differentiated between three types of teams, each with a different level of collaboration: traditional small teams collaboratively designing a course, leader-based teams in which one teacher has oversight over the team and creates connections between various instructors, and modular teams that divide courses into small parts, where each teacher covers his or her own part. According to Bryant et al., traditional and leader-based teams require a medium to high level of member commitment, whereas modular teams need less interaction between team members and therefore also need less commitment.

##### Self-efficacy

Teachers who lack self-efficacy—in this case, have a low rating of their own ability to organize and execute new teaching strategies or methods—can be scared of changing their teaching practices. According to the qualitative studies of Margalef García (2011), Margalef García and Roblin (2008), and Schuck et al. (2013), teachers face uncertainty as a result of innovation processes. Margalef García and Roblin (2008) described how for some teachers, “deep unbalances and questioning about [the] ways of perceiving teaching and learning were generated when [teachers] faced a methodological approach based on principles, beliefs and values which demanded a reconstruction of their knowledge and practice” (p. 114). Therefore, when confronting this insecurity, teachers needed to build new self-efficacy to be successful in building new strategies and practices. Teachers only embraced the uncertainty and took the challenge of exploring its potential when they were able to witness the impact of the new strategies on student learning and the results obtained by their colleagues (Roblin & Margalef, 2013).

##### Professional identity

The professional identity of teachers in higher education can also influence their participation in a professional development intervention. According to interviews and video recordings of seminars of Blanton and Stylianou (2009), teachers’ professional identity—in their study called faculty identity—in higher education stems more from the content or their discipline than from their teaching of this discipline. Participating in a team-based professional development intervention, such as a community of practice when having a research-focused professional identity, may require an identity shift by the participants. Blanton and Stylianou (2009) stated that participants whose identity is that of a content expert must undergo an identity shift and become a teaching scholar again, which can be a great challenge when building communities of practice in higher education. Furthermore, Margalef García and Roblin (2008) criticized the individualism that is predominant in higher education when it comes to teaching. An identity shift from being an isolated researcher/teacher to becoming a more team-oriented teacher is needed to make team innovations a success and ensure team-based professional development of teachers.

##### Availability

Teams that have been working together for a longer period of time, maybe even years, can experience a disruption in their team process and practices when a team member leaves for some period of time. Teachers in the mixed-methods study by Bakah et al. (2012) described how their participation in design teams was affected by long term study leave or sabbatical leave. Dickerson et al. (2014) pointed out that teachers experienced constraints in the form of team members being away. However, neither study went into further detail on how the team was affected by this.

#### Conditions at the Team Level

Five conditions were found at the team level: team interaction, goals and objectives, team composition, team leadership, and small group work.

##### Team interaction

When it comes to working in teams, the interactions taking place between team members are of great importance for the success of a team. First, trust within the team in very important for successful team-based professional development. Establishing effective relationships between team members is said to be crucial for the development of trust (Bryant et al., 2014; Harwood & Clarke, 2006; Margalef García, 2011; Norton et al., 2011). In the literature, face-to-face interactions were described as playing an important role when it comes to building trust. Teams that exist partially or fully online therefore often face trust issues (e.g., Houghton et al., 2015; Keevers et al., 2014). Keevers et al. (2014) described in their mixed-methods study the need for more face-to-face communication in order to strengthen the relationships between team members.

Second, another aspect of team interaction, closely related to trust is the concept of team cohesion that can be crucial for a team-based professional development intervention as well (e.g., Margalef García, 2011). According to Norton et al. (2011), by deepening their level of mutual support teachers connected with each other. However, they also stated that developing this connection between new teams is critical but challenging to achieve. Likewise, Schuck et al. (2013) described how teachers in their qualitative study were not able to form a cohesive professional learning community because their team was too large and diverse to operate cohesively.

Finally, the communication within a team, meaning the open information sharing between team members inside and outside of formal meetings, is an important part of team interaction and can also play an important role for a team-based professional development intervention (e.g., Dickerson et al., 2014). Bryant et al. (2014) found during their focus group interviews that teams were successful when there was open communication among the team members. Harwood and Clarke (2006) described communication, along with the willingness to take risks such as sharing information, as processes supporting team development. In the mixed-methods study by Keevers et al. (2014), participants stressed the importance of direct face-to-face communication.

##### Goals and objectives

Another influential factor at the team level was the clarity of goals and objectives set for the team (Margalef García, 2011; Roblin & Margalef, 2013; Schuck et al., 2013). Roblin and Margalef (2013) described in their qualitative study how teachers in teams face the dilemma of either pursuing their own goals and interests, or balancing their own goals and the goals of the community. Furthermore, Schuck et al. (2013) described in their article how several team members left a professional learning community because they lacked (among other things) shared goals and practice.

##### Team composition

The composition of a team in terms of team heterogeneity and the involvement of external parties can be of influence as well. Team heterogeneity can be an important factor for team-based professional development (Deni & Malakolunthu, 2013; W. Green et al., 2013; Keevers et al., 2014; Kosnik et al., 2015; Schuck et al., 2013). According to the qualitative studies of Kosnik et al. (2015) and Blanton and Stylianou (2009), heterogeneous teams have more to offer, as experienced teachers (old-timers) and inexperienced teachers (newcomers) can learn from each other. However, being an old-timer or newcomer does not depend on seniority or academic experience but the time spent as a member of the community of practice (W. Green et al., 2013). Keevers et al. (2014) stated that a status difference based on formal qualifications in relation to research could influence peer-to-peer interactions, as less experienced participants might be reluctant to approach experienced academics. Diversity can also have a negative effect on teams, as great diversity within the team can hinder cohesive functioning (Schuck et al., 2013). However, in their article, the authors did not specifically define the nature of the diversity to which they were referring.

Involving other people, such as colleagues from outside the team, which changes the team composition of a team, can be beneficial for teams as well. In the qualitative study by Dickerson et al. (2014), teachers identified involving others such as students, technicians, or critical friends as a good practice applied in their team-based professional development intervention. However, most articles that mentioned third-party involvement argued for an external facilitator of group or learning processes. According to Margalef García (2011), teams need the support of an external expert, who can make teachers aware of their tacit theories and help adjust them. Margalef García (2011) describes in her qualitative study that a facilitator needs to be involved in a team, to foster understanding about problems and their alternatives. Blanton and Stylianou (2009) argued based on their qualitative study that professional development interventions should be organized and led by more experienced faculty—a “more knowing other” (p. 88)—because teachers were unable to ask their busy peers for help or support.

##### Team leadership

Leadership within the team can play an important role in the success of a team-based professional development intervention as well. Excellent leadership was identified as an effective practice in the qualitative study by Dickerson et al. (2014). However, what this excellent leadership entailed was not specified. Likewise, Margalef García (2011) indicated that the leadership shown by group members within a teacher learning community or the leadership of the team’s coordinator is important for the community’s progress. Margalef García (2011) argued for the strengthening of “peer coaches” or distributed leadership within a team, as this contributes to enhanced development as well as independence in the teams. In comparison, in their mixed-methods study, Harwood and Clarke (2006) argued for a team leader or “teaching champion” who facilitates communication within the team to break down barriers and implement change.

##### Small group work

Another success-related factor mentioned in several studies is small group work, which means that teams temporarily split up into smaller groups to work on a specific task and bring back their results to the whole team afterward. In the qualitative study by Dickerson et al. (2014), participants identified small group work as a good practice. Poyas and Smith (2007) stated the importance of discussions in small groups for the reshaping of practical issues and professional definition. The participants wanted to exchange experiences in a safe environment that was provided in small group settings.

#### Conditions at the Organizational Level

Four conditions were found at the organizational level: organizational support, rewards, research focus, and finances and resources.

##### Organizational support

The support of the organization in which the team-based professional development intervention takes place is of great importance as the organization allocates the resources the participants need to successfully participate in the intervention. Organizational support can take various forms such as explicit time allocated for participation and recognition for team efforts and achievements.

Time can be a challenging factor for team-based professional development (Margalef García, 2011; Margalef García & Roblin, 2008). Teachers need time to master and apply new operational methods and tools (Deni & Malakolunthu, 2013). Moreover, teams often find it difficult to find the time to meet or finish a training (Bakah et al., 2012; Dickerson et al., 2014; Houghton et al., 2015; Rienties, Brouwer, & Lygo-Baker, 2013; Schuck et al., 2013). Harwood and Clarke (2006) noted that time and space for teachers are important processes supporting continuous professional development. Both Bakah et al. (2012) and Kosnik et al. (2015) identified heavy workload as a challenge for team-based professional development interventions.

In order to ensure the sustainability of team-based professional development interventions, the teams need to be supported by the management. Teachers require recognition and encouragement of team activities by the management (Bakah et al., 2012; Harwood & Clarke, 2006; Keevers et al., 2014; Kosnik et al., 2015; Margalef García, 2011). Teams must be incorporated in the structure and strategic plan of the higher education institution and need to be better managed by the institution (Bakah et al., 2012). Participants in the mixed-methods study by Poyas and Smith (2007) stated that they felt that their superiors had almost no idea of how successful they were and that they had little support when there were problems.

##### Rewards

Another factor that can influence teams or teachers’ participation in team-based professional development interventions is whether participation in these teams is not only recognized but also rewarded by the institution (e.g., Dickerson et al., 2014; Keevers et al., 2014). Blanton and Stylianou (2009) stated in their qualitative study that “while there might be a perception that teaching matters at a particular institution, the question remains as to how that perception translates into systemic reward of teaching excellence” (p. 85). Bryant et al. (2014) argued based on focus group interviews that the university administration should provide incentives and recognition for developing and implementing interdisciplinary or other collaborative courses which must be developed in teams. Monetary rewards for team members were refused, as teachers believed that financial rewards for team members could hinder upscaling and sustainability within the institution and that some teachers might only participate in teams for the financial benefits, undermining team activities (Bakah et al., 2012). Instead, team accomplishments should be taken into account when deciding about teacher promotion (Bakah et al., 2012). Up to this point, teachers felt that participating in team activities could diminish their chances of getting tenure (Bryant et al., 2014; Houghton et al., 2015).

##### Research focus

Another influential factor is the university’s research focus. As teachers at universities are mostly rewarded for their research output instead of their teaching excellence, Bryant et al. (2014) stated that the university’s focus on research might distract from its teaching mission. Participants in their focus group interviews also mentioned that they felt that people in higher administrative positions did not favor innovative teaching (e.g., collaborative teaching), as it takes time away from research. On the other hand, Rienties, Brouwer, and Lygo-Baker (2013) indicated in their quantitative study that their online training with a team component was especially attractive for teachers from research-intensive universities, as they were not yet familiar with effective technology integration in their teaching.

##### Finances and resources

Team-based professional development interventions are also influenced by the resources provided by the institution, for example, in the form of funding (e.g., Kosnik et al., 2015). Participants in the focus group interviews of Bryant et al. (2014) claimed that many decisions about teaching missions are based on a fiscal perspective instead of a pedagogical perspective, resulting in a lack of resources allocated to innovative teaching methods. Teaching is said to become a number game, which does not ensure high teaching quality.

## Discussion

Although working in teams and collaborating with colleagues has already become more common in primary and secondary education in the past several years, team-based professional development in higher education is just emerging. Little is therefore known about the possible advantages of working in teams in higher education. This review was conducted to provide an overview of these advantages in the context of higher education. The research questions guiding this review were the following: What are the benefits of team-based professional development in higher education in terms of (a) teacher attitudes and (b) teacher learning, and (c) under what conditions are these interventions most successful?

### The Effect on Teacher Attitudes

First, when looking at the impact of team-based professional development interventions on teachers’ attitude, it is evident that attitude changes in this context have not really been studied yet. The variables attitudes as an outcome of a team-based professional development intervention therefore had to be dropped from further analysis. Instead of describing attitudes, various studies either focused on teacher satisfaction with the intervention (e.g., Bakah et al., 2012; Keevers et al., 2014; Poyas & Smith, 2007; Rienties, Brouwer, Bohle Carbonell, et al., 2013) or described enhanced teacher confidence due to the intervention (e.g., Dickerson et al., 2014; Rienties, Brouwer, Bohle Carbonell, et al., 2013; Rienties, Brouwer, & Lygo-Baker, 2013).

### The Effect on Teacher Learning

Second, most articles in this review studied the effect of team-based professional development interventions on teacher learning in the form of new knowledge and skills gained. Several categories of teacher learning could be identified that were affected by these interventions. Teachers reported having improved their collegiality by working closely together with them in a team, which, for example, led to a greater awareness about similarities and common interests between themselves and their colleagues. Some teachers even reported that their colleagues became role models for them. Furthermore, teachers learned to critically reflect on their teaching practice. Several studies also described how teachers adapted their teaching approach due to their participation; for example, their teaching became more student-centered. To achieve this, teachers reported that they learned effective teaching practices that involved concrete examples of what works in the classroom.

Participating in a team-based professional development intervention also had a positive effect on teachers’ pedagogical knowledge. Teachers began to experiment with new ideas, which boosted their confidence if they were successful. Another effect of team-based professional development interventions in higher education involved the student–teacher relationship. Several studies (Deni & Malakolunthu, 2013; Dickerson et al., 2014; W. Green et al., 2013; Harwood & Clarke, 2006; Norton et al., 2011) reported that by participating in these interventions, teachers gained a better understanding of students and how to support them. Finally, teachers gained a better understanding of their identity as a teacher.

When comparing these findings to the literature on teacher learning in teams in other educational contexts, there is much consensus across the different educational contexts. For example, Vangrieken et al. (2015) reported in their review study that teachers in secondary education also experienced improved collegiality by participating in teams. Furthermore, a shift to a more student-centered teaching approach and an extension of teachers’ repertoire with regard to teaching activities and tools were also mentioned (Vangrieken et al., 2015). For example, Meirink, Meijer, and Verloop (2007) found that teachers in secondary education who were participating in collaborative groups were experimenting with either a teaching method adjusted from that of a colleague, a teaching method copied from that of a colleague, a self-invented teaching method, or a teaching method that was developed in a group meeting.

Although when it comes to learning there is no big difference in categories of learning between higher education and other educational contexts, several of these categories might play a different role in higher education than in other educational contexts. As many teachers in higher education have had little teacher training, their pedagogical knowledge and skills might need more attention, as well as their perception of themselves as teachers. Therefore, effects on one’s teaching approach, teacher identity, and pedagogical knowledge might play a different role in a higher education context. Furthermore, as teachers in universities are also researchers, getting to know their colleagues may also have a positive effect on their research by opening up new collaboration opportunities.

### Factors Influencing Professional Development in Teams

Finally, it was possible to identify several factors supporting team-based professional development. All of these factors can be organized according to three levels: individual teacher level, team level, and organizational level.

#### Factors at the Individual Level

Factors at the individual level include teacher attitudes, teacher motivation, commitment, teachers’ self-efficacy beliefs, their professional identity, and teacher availability. When comparing these factors found in higher education to factors found in other educational contexts, it appears that several of these factors are applicable to all educational contexts. Teacher attitudes and commitment play a role in other educational contexts as well, as found in the studies by Stoll, Bolam, McMahon, Wallace, and Thomas (2006) and Vangrieken et al. (2015). Furthermore, Binkhorst et al. (2015) identified teacher motivation to participate in a team also to be important in secondary education. Velthuis, Fisser, and Pieters (2015) found that the self-efficacy of primary teachers participating in a teacher design team played a crucial role as a motivational factor.

Therefore, teacher attitudes, motivation, commitment, and self-efficacy can play an important role in other educational settings as well. There are however some factors that are specific for higher education, or are important in a different way in other educational contexts. These factors are teacher identity and teacher availability. In higher education, teachers are mostly researchers first and teachers second, meaning that their focus is primarily on their research activities instead of teaching students, which influences their professional identity as well as their availability for teaching. Furthermore, in higher education, it is quite common for researchers to take a sabbatical leave or go to other universities as visiting scholars for some time. This can affect team processes as essential parts of the core team might be missing for some time and come back later. Taking a sabbatical leave or visiting other universities might also have a positive effect on teacher learning and teacher attitude changes within a team, as those teachers might return with new input to the team.

#### Factors at the Team Level

Factors at the team level include team interaction, the clarity of the teams’ goals and objectives, the team composition, team leadership, and small group work. When comparing the findings of this review study to findings in other educational contexts, all factors again seem to be equally important in all educational contexts. Team interaction, for example, is described in several studies in other educational contexts (e.g., Bryk, Camburn, & Seashore Louis, 1999; Eameaim et al., 2009; Vangrieken et al., 2015). Furthermore, Binkhorst et al. (2015) and Vangrieken et al. (2015) found that shared goals as well as team composition also play an important role in other educational contexts. For example, they both described a teacher’s right to make decisions about their own teaching practice. Additionally, Stoll et al. (2006) described the need of schools to seek help of external agents for their professional learning communities. Both, Stoll et al. (2016) and Vangrieken et al. (2015) stressed the role of team leadership as a facilitating factor for team-based professional development interventions. Moreover, small group work could also have an impact on teams in non–higher education contexts as team size has been found to facilitate collaboration in teams (Vangrieken et al., 2015). As all of these factors at the team level found in higher education can also be found in other educational contexts, there seems to be more general applicability of these factors for successful teacher professional development across contexts.

#### Factors at the Organizational Level

Factors at the organizational level that are mentioned in the literature are the organizational support in terms of time, workload and support of the management, recognition of participation, the university’s research focus, and the finances and resources allocated to the teams. When comparing these factors to factors found in other educational contexts, again there is some consensus across contexts. For example, Binkhorst et al. (2015), Stoll et al. (2006), and Vangrieken et al. (2015) all stressed the importance of time and school support for teacher teams. Bryk et al. (1999) specifically describe the supervision and leadership of the principal as an important facilitating factor for professional communities.

However, there are also some factors at the organizational level that were found in our review that might be less of an issue in these other contexts. One of these factors is the research focus of research intensive universities, which can distract from the universities’ teaching mission. As research is only a primary focus in higher education, this factor is higher education–specific. Another factor that is connected to this research focus is the distribution of resources between research and teaching, especially at research-intensive universities. Finally, although recognition of participation in team-based professional development interventions can be an issue in other educational contexts as well, this issue can be greater in the context of universities, as participation in these interventions might even have a negative impact on teachers’ possibilities of promotion. Participation in teams leaves less time for research, which results in less research output, which can prevent teachers from getting promoted.

**Figure 3:**
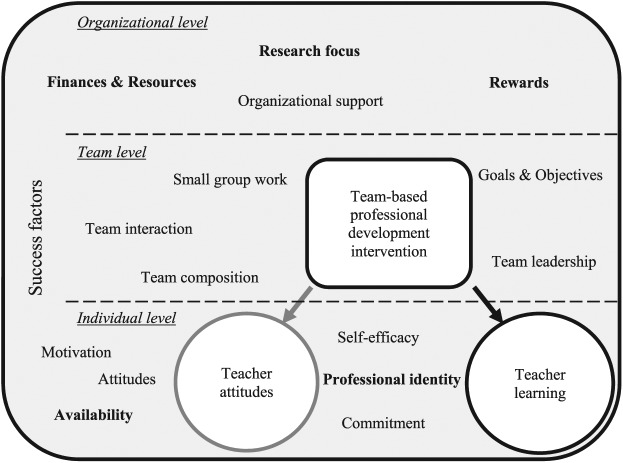
Factors influencing professional development in teams at each level. *Note*. Higher education–specific factors are printed in bold letters.

Overall, it can be said that there is much consensus between higher education and other educational contexts when it comes to teacher learning and factors that influence the professional development of teachers in team-based interventions. Although there seem to be factors that are more generally applicable to almost all educational contexts, there are also factors at the individual and organizational level that seem to be of unique influence in higher education, and especially in universities with a strong research focus. In Figure 3, an overview is given of all factors influencing teacher professional development in teams in the context of higher education, as mentioned by at least two articles in this review. All factors that are higher education–specific or might play a bigger role in higher education than in other educational contexts, as explained above, are printed in bold letters.

### Limitations

There are several limitations which have to be acknowledged. First, there are not many articles published on team-based professional development interventions in higher education. To provide an overview of the existing research, we included various types of team-based professional development interventions in this review. However, these teams are comparable, as they all have a strong focus on curriculum development and/or teacher learning. For those studies focusing on teacher teams as part of a larger formal professional development intervention, it is also difficult to determine the impact of the teamwork on teacher learning compared to the other parts of the intervention. Second, there was a clear focus on qualitative research methods among the studies included in this review, with a preference for interview studies. These studies provide valuable in-depth information on the topic but were mostly conducted in the form of case study designs, which are often less generalizable.

Third, the studies that have been published on the topic of team-based professional development in higher education often did not meet our quality criteria, as they lacked, among other things, a sufficient description of the methods used and a description of the data analysis. Fourth, some articles remained less specific when it came to reporting about results, mostly when it came to learning outcomes or lacked clear definitions of the concepts described. However, we wanted to include these articles because they give us valuable firsthand information that other studies cannot. Fifth, we were not able to study links between individual, team and organizational factors, as this did not lie within the scope of this article. Finally, when it comes to individual factors influencing teacher learning, we have not distinguished between teacher beliefs and teacher practice, because of the self-report nature of the data (see Fang, 1996; Mansour, 2013). However, this review has provided an overview of the effects of team-based professional development interventions on teacher learning in higher education as well as influential factors in this regard, which can be used as a starting point for further research.

### Implications for Further Research

There is a great need for large-scale quantitative studies along with more in-depth qualitative studies on the topic of effects of team-based professional development interventions in higher education on teacher learning, as well as studies of success-related factors in these interventions. It is also desirable to conduct more mixed-methods studies that combine large-scale quantitative data and in-depth qualitative data. Research is needed about the effects of team-based professional development interventions, especially concerning the effects on teacher attitudes. Furthermore, although some conditions for successful professional development in teams have already been identified, the question remains whether there are additional conditions and whether all conditions are equally important. Moreover, researchers need to be more precise when reporting on teacher learning outcomes, especially when it comes to improvement in teacher knowledge and skills due to participation in these teams. For further research, authors need to make sure that they provide crucial information to ensure reliable added value for knowledge building in this field. Most studies remained fairly general when it came to reporting on learning outcomes. Articles often did not go into detail about these learning outcomes, for example, only stating that new strategies were learned without specifying the nature of these practices. Furthermore, it would be interesting to further study the links between the individual, team, and organizational factors to get an understanding of how these factors are related to each other. Finally, additional research on the individual-level factors as well as learning outcomes, especially the effects on teachers approach to teaching is needed, which specifically focusses on the differences between teacher beliefs and teacher practice.

### Implications for Practice

This review has shown that team-based professional development interventions can be successful in fostering teacher learning in higher education. Furthermore, this review has shown that a university may want to give special attention to influential factors at the individual teacher level, team level, and organizational level when trying to implement a professional development intervention that involves teachers collaborating in a team. For example, the university must create an environment that enhances teacher learning and positively influences the teachers’ attitudes. To do so, universities would, for example, need to provide extra time for teachers to spend on the team intervention if extra workload is created by participating in a team. It is also very important to reward teachers for their team achievements, by taking their successes into account during promotion and in the tenure process. Universities need to value innovative teaching as well as research achievement. Therefore, universities can do a lot at the managerial level to support teacher professional development in teams. By doing this, universities can promote higher quality teaching and ensure that students are better prepared for the changing job market.

## Notes

This article was funded by the 4TU Centre for Engineering Education.
